# Matricellular Proteins: A Sticky Affair with Cancers

**DOI:** 10.1155/2012/351089

**Published:** 2012-02-09

**Authors:** Han Chung Chong, Chek Kun Tan, Royston-Luke Huang, Nguan Soon Tan

**Affiliations:** School of Biological Sciences, Nanyang Technological University, 60 Nanyang Drive, Singapore 637551

## Abstract

The multistep process of metastasis is a major hallmark of cancer progression involving the cointeraction and coevolution of the tumor and its microenvironment. In the tumor microenvironment, tumor cells and the surrounding stromal cells aberrantly secrete matricellular proteins, which are a family of nonstructural proteins in the extracellular matrix (ECM) that exert regulatory roles via a variety of molecular mechanisms. Matricellular proteins provide signals that support tumorigenic activities characteristic of the metastastic cascade such as epithelial-to-mesenchymal (EMT) transition, angiogenesis, tumor cell motility, proliferation, invasion, evasion from immune surveillance, and survival of anoikis. Herein, we review the current understanding of the following matricellular proteins and highlight their pivotal and multifacted roles in metastatic progression: angiopoietin-like protein 4 (ANGPTL4), CCN family members cysteine-rich angiogenic inducer 61 (Cyr61/CCN1) and CCN6, osteopontin (OPN), secreted protein acidic and rich in cysteine (SPARC), tenascin C (TNC), and thrombospondin-1 and -2 (TSP1, TSP2). Insights into the signaling mechanisms resulting from the interaction of these matricellular proteins and their respective molecular partner(s), as well as their subsequent contribution to tumor metastasis, are discussed. In addition, emerging evidences of their promising potential as therapeutic options and/or targets in the treatment of cancer are also highlighted.

## 1. Introduction

Cancer research has generally focused on cell-autonomous behavior and the molecular genetics of malignant cells. Malignant tumors, however, are more than a mere mass of proliferating cancer cells. Tumors are highly complex structures comprising a plethora of cell types and oncogenic secretory factors and are structurally supported by the extracellular matrix (ECM). In addition, cancer cells modulate various cellular functions and participate in heterotypic interactions via secreted factors to aid in growth and metastasis. These interactions usually set off a cascade of downstream molecular signaling events that determine the outcome of a malignancy.

**Figure 1:**
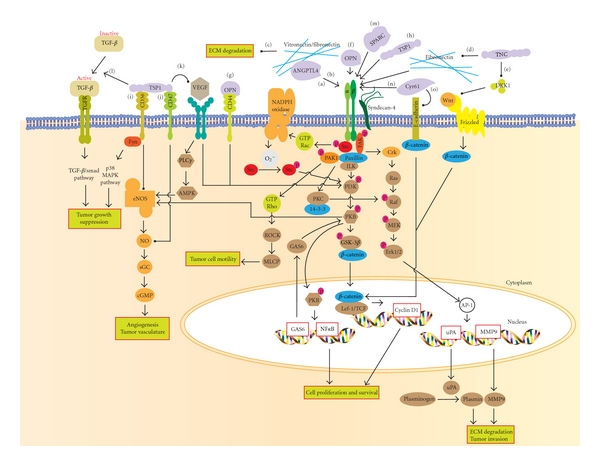
Summarized the signaling mechanisms of various matricellular proteins contributing to cancer progression. ANGPTL4 binds to both integrins and ECM to promote tumor survival, tumor invasion and modulate the availability of ECM. (a) ANGPTL4 interacting with integrin activates Rac1 and NADPH oxidase, which generate high level of O_2_^−^. This will further activating the Src machinery and stimulates its downstream PI3K/PKB mediated survival pathway. (b) ANGPTL4 interacting with integrin also activates FAK-src-PAK1 signaling and PKC/14-3-3 mediated pathway which modulate cell migration via integrin internalization. (c) ANGPTL4 binds specific matrix proteins and delays their degradation by proteases. However, this association does not interfere with integrin-matrix protein recognition unlike TNC. (d) TNC can compete with fibronectin to bind integrin *α*5*β*1 coreceptor, syndecan-4, which blocks the activation of promigratory FAK/RhoA/ROCK signaling pathway. (e) TNC can activate Wnt signaling by downregulating the soluble inhibitor DKK-1, thus resulted in nuclear localization of *β*-catenin. Nuclear *β*-catenin interacts with TCF/LEF to promote the expression of genes contributing to tumor formation, survival, and metastasis. OPN can interact with several (f) integrins and also (g) CD44 family of receptors. These complexes are able to mediate tumor cell survival through PI3k/PKB pathway activation and motility for detachment or invasion of tumor cell through the activation of AP-1-dependent gene expression via the MEK/Erk pathway. (h) Certain domains of TSP1 (such as NoC1) can bind directly to integrins to activate signaling proteins such as Erk1/2 and paxillin which modulates tumor formation. (i) TSP1 binding to CD36 activates Fyn and p38 MAPK pathway which is essential for the suppression of tumor growth. (j) TSP1 can also bind CD47, to modulate sGC and cGMP-dependent protein kinase, thus inhibiting the NO signaling necessary for angiogenesis. (k) TSP-1 association with CD47 or direct competitive binding of TSP1 to VEGF can inhibit VEGFR2 signaling. VEGFR2 activates the PI3K/PKB pathway which leads to activation of eNOS/NO signaling. Simultaneously, VEGFR2 can also signal through PLC*γ*, which further increases AMPK-mediated eNOS phosphorylation and NO production. eNOS/NO signaling regulate downstream targets that increase endothelial cell proliferation, migration, survival, and permeability. (l) TSP1 can activate TGF*β*/smad pathway to inhibit tumor cell proliferation and induce apoptosis. (m) SPARC binds integrin, inducing ILK/FAK/PKB activation to increase cell migration. (n) Cyr61 can promote tumor cell proliferation and survival through the activation of integrin mediated signaling pathway either by direct binding with integrin or integrin-syndecan4. The downstream intracellular events may be mediated through the FAK/PI3K/PKB signaling pathway, resulting in either activation of the NF-*κ*B survival pathway or phosphorylation of GSK3*β* and nuclear translocation of *β*-catenin for cell proliferation. (o) Cyr61 allows protein degradation of E-cadherin leading to *β*-catenin translocation.

**Figure 2:**
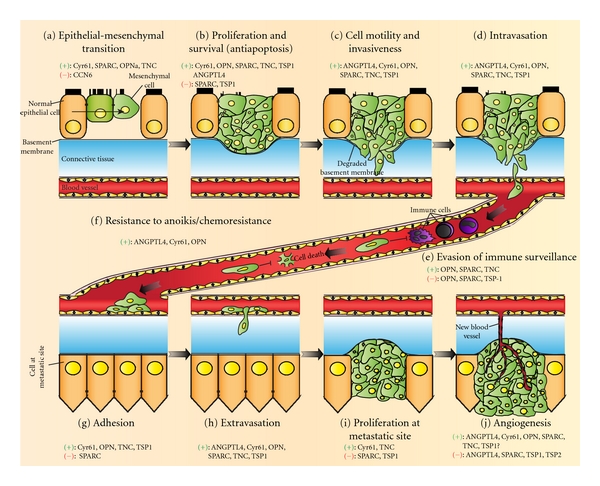
Schematic illustration of cancer progression from primary tumor to metastasizing cancer and the involvement of various matricellular proteins in each process. Aberrant expression of matricellular proteins in tumors or in the surrounding stromal cells induces or inhibits the following tumorigenic and cancer progression events. (a) Epithelial-to-mesenchymal transition allows a normal epithelial cell, which normally adheres to basement membrane, to undergo a series of cellular and biochemical changes (i.e., a switch from E-cadherin to N-cadherin and increased vimentin expression) to adopt a mesenchymal phenotype. (b) Promotion of cell proliferation and survival in tumor cells lead to uncontrolled tumor growth. (c) Secretion of matrix metalloproteinases by tumor cells and acquisition of tumor cell motility result in basement membrane degradation and the increased invasiveness of the tumor cells. (d) Intravasation of invasive cancer cells through the basal membrane and endothelial monolayer allows the cancer cells to invade into the circulation. (e) Diminished immune surveillance and leukocyte recruitment against the circulating cancer cells permit the cells to survive in the circulation. (f) Matricellular proteins also promote resistance against anoikis and chemotherapy in order for the cancer cells to survive in the circulation. (g) Interactions of the matricellular proteins secreted by cancer cells with the surface receptors on endothelial cells result in an intermediate cell adhesion that allows the cancer cells to dock on the endothelial monolayer. (h) Adhered cancer cells subsequently undergo trans-endothelial migration through a process called extravasation to invade a distant site. (i) Establishment of new tumors at the metastatic site is dependent on the proliferation of invaded cancer cells; (j) Neovascularization within the tumor mass via angiogenesis is crucial for tumors to grow beyond a certain size. (+) and (−) denote positive and negative effects, respectively, imposed by the indicated matricellular proteins on the selected events. The disparate functions of any given matricellular proteins are dependent on the cell-type context and the specific structural domains that are expressed.

 Tumor metastasis is a multistep process involving the acquisition of malignant cell phenotypes that allow cancer cells to leave the primary tumor site and form secondary metastases via blood circulation (Figure 1). Each of these steps involves the cointeraction and coevolution of the tumor and its microenvironment and is in part affected by the heterotypic interactions between the cancer cells and neighboring stromal cells [1]. The tumor microenvironment consists of a myriad of cellular components, such as the non-malignant stromal fibroblasts, and endothelial cells, and an ECM comprised of proteins with structural and regulatory functions, including collagen, fibronectin and matricellular proteins [1, 2]. Matricellular proteins are a group of structurally diverse, ECM-associated glycoproteins, that are secreted by tumor and neighboring stromal cells in the tumor microenvironment [3, 4]. They have regulatory roles, such as the modulation of cell-cell and cell-matrix interactions, but do not contribute significantly to the structure of the ECM [4]. These proteins facilitate and contribute to various aspects of cancer cell behavior and growth, such as epithelial-mesenchymal transition (EMT), angiogenesis, cell proliferation and survival, as well as motility and ECM degradation (Figure 1) [2]. Numerous studies have shown how their interactions with the various cellular components initiate downstream signaling events that culminate in the acquisition of various hallmarks of cancer (Figure 2) [5].

 In this review, we focus on six different matricellular proteins-angiopoietin-like protein 4 (ANGPTL4), CCN family members cysteine-rich angiogenic inducer 61 (Cyr61/CCN1) and CCN6, osteopontin (OPN), secreted protein acidic and rich in cysteine (SPARC), tenascin C (TNC), and thrombospondin-1 and -2 (TSP1, TSP2)—highlighting their roles in metastatic progression. Although the growing family of matricellular proteins consists of other members of the small integrin-binding ligand N-linked glycoproteins (SIBLINGs), lipocalin, and galectins, among others, their roles in cancer have not been extensively studied and shall be reserved for future reviews [2]. As tumor metastasis is a major hallmark of cancer progression and usually indicates a poor prognosis for the patient, this review discusses the role and contribution of these six matricellular proteins in the various steps of the metastatic process. Furthermore, this review will discuss the signaling pathways triggered by the interaction of these matricellular proteins with their respective molecular partner(s) (Table 1) and their subsequent contribution to tumorigenesis and metastasis (Figure 1).

## 2. Epithelial-Mesenchymal Transition

Epithelial-Mesenchymal Transition is an important biological process during embryonic development. During this process, polarized epithelial cells, which are normally tightly joined together through intercellular junctions and adhered to the basal membrane, undergo multiple biochemical changes that enable the cells to acquire mesenchymal, fibroblast-like properties. EMT is characterized by the disruption of cell-cell adherence mediated by E-cadherin, the loss of apical-basal polarity, increased cell motility, cytoskeleton reorganization and matrix remodeling through the production of ECM components, such as fibronectin and type I collagen [6]. Several transcription factors have been implicated in the repression of E-cadherin, including zinc-finger proteins of the Snail (Snai1)/Slug (Snai2) family, *δ*EF1/ZEB1, SIP1, and the basic loop-helix E12/E47 factor (see review [7]). Accumulating evidence has indicated the occurrence of EMT-like events during tumor progression and malignant transformation, thereby conferring the incipient cancer cells with invasive and metastatic properties [6, 7]. In fact, a high-throughput study in melanoma identified EMT as a major determinant of metastasis [8]. EMT is now recognized as a potential mechanism for carcinoma progression and a determinant of tumor staging. Several matricellular proteins, such as SPARC, OPN, CCN1, CCN6, and TNC, have been implicated in either the promotion or suppression of EMT, highlighting their role in cancer progression and malignant behavior of cancer cells.

SPARC, also known as osteonectin, is a secreted glycoprotein and the prototypical member of a family grouped on the basis of an extracellular calcium-binding module. The ectopic expression of SPARC in normal melanocytes induces a fibroblast-like morphology characterized by reduced epithelial markers on the transcript level (e.g., E-cadherin and Mucin-1) with a concomitant increase in mesenchymal markers, such as vimentin, fibronectin, and Lef-1 [9]. Moreover, these transfected cells exhibit a strong reduction in P-cadherin expression, which was recently found to promote cell-cell adhesion and counteract invasion in human melanoma [9, 10]. The overexpression of SPARC also stimulates melanoma cell invasiveness mediated by the phosphorylation of focal adhesion kinase (FAK) and Snail-induced repression of E-cadherin promoter activity [11]. Integrin-linked kinase (ILK) interacts with integrin subunits *β*1 and *β*3 in focal adhesion complexes and has been known to be a key regulator of multiple signaling pathways [11]. SPARC modulates ILK activity through direct protein-protein interaction, which may be the mechanism responsible for the loss of E-cadherin and cell adhesion [12]. SPARC-induced cell migration is likely to involve a mechanism independent from the activation of the Arg-Gly-Asp (RGF)-binding integrins *α*v*β*3 and *α*v*β*5, as evidenced by the lack of an RGD sequence in the SPARC protein [13]. These observations suggest that aberrant SPARC overexpression induces EMT and the loss of intercellular adhesion, thus enhancing cell migration, invasiveness and metastatic capacity.

 Osteopontin (OPN) is a secreted transformation-associated phosphoprotein and has been implicated in tumorigenesis. The upregulation of OPN expression has also been reported in a variety of human cancers, such as breast, prostate, nonsmall cell lung cancer (NSCLC), and colon carcinomas [14]. Importantly, several studies have suggested a relationship between OPN levels and the progression of these cancers [15–18]. Furthermore, the expression of OPN is increased in NSCLC, and the overexpression of different OPN isoforms in NSCLC cell lines reveals functional heterogeneity associated with the individual isoform expressed [19]. The ectopic expression of OPNa isoform results in an increased expression of mesenchymal markers, including MMP-2, MMP-9, Snail-1, Snail-2, N-cadherin, ILK, and vimentin, with a concomitant downregulation of epithelial markers, such as E-cadherin, desmoplakin, and cytokeratin 18 and 20 [19]. These data may partly explain the malignant behavior of NSCLC cells as a result of EMT. Interestingly, OPNa overexpression triggers EMT pathways and malignant behavior *in vitro*, whereas OPNc overexpression results in a decrease in the same properties. The only difference between these isoforms is the transcription of exon 4, which is a 27 amino acid sequence at the N-terminus of the protein [19]. This suggests that exon 4 may act as an important regulator of NSCLC malignant potential [19].

 The CCN family of cysteine-rich matricellular proteins contains six members in vertebrates [20]. CCN1 or Cyr61, in particular, is known to play important roles in cell adhesion, proliferation, migration, differentiation, and angiogenesis during normal developmental and pathophysiological processes [21]. An aberrant overexpression of Cyr61 has been reported in various human cancers, including gliomas, pancreatic ductal adenocarcinoma (PDAC), prostate, and breast cancers [22–25]. Earlier reports also show that higher levels of Cyr61 protein are associated with advanced breast adenocarcinoma, PDAC, and gliomas, which suggest its involvement in cancer progression and metastasis [22, 23, 25]. Cyr61 has been found to play a critical role in pancreatic cancers and aggressive PDAC cell lines through the induction of EMT and the expression of mesenchymal/stem cell markers; additionally, stem cell-like Cyr61 silencing reduces the aggressive behaviors of malignant cells by obliterating the interlinking pathological events, such as EMT reversal, blocking the expression of mesenchymal traits, and inhibiting migration [23]. In contrast, CCN6 exhibited inhibitory effects on breast cancer growth and invasion. CCN6 protein levels are reduced in invasive carcinomas T with lymph node metastasis [26–28]. Notably, the downregulation of CCN6 promotes EMT and invasion in nontumorigenic breast epithelial cells by upregulating mesenchymal proteins and decreasing epithelial proteins [27, 29]. The molecular basis of CCN6-mediated EMT in mammary epithelium likely involves the induction of Snail and ZEB1 transcription levels and the subsequent inhibition of E-cadherin promoter activity [27]. The administration of exogenous recombinant CCN6 protein can impede the activation of the insulin-like growth factor (IGF-)1 pathway and lead to a reduction in ZEB1 expression, suggesting that the CCN6-mediated inhibition of ZEB1 transcription is dependent on the attenuation of the IGF-1 signaling pathway [27, 30].

 Tenascin-C (TNC) was first discovered as a protein in the stroma of gliomas and as a myotendinous antigen in connective tissues [31]. TNC is the founding member of a group of secreted matricellular glycoproteins consisting of the tenascins-X, -R, -Y, and -W [31]. The increased expression of TNC in glioma, breast, and colon cancers has been correlated with a poor survival prognosis [32, 33]. Recently, the expression of TNC was found to be elevated in advanced melanomas and in the stem cell-like side populations of the melanoma spheres, suggestive of a role in cancer stem cells [34]. The administration of exogenous TNC protein to the MCF-7 breast cancer cells induces an EMT-like phenotypic change accompanied by a delocalization of E-cadherin and *β*-catenin from cell-cell contacts [35]. The EMT phenotype was accompanied by the activation of Src and FAK that are localized with *α*v integrin-positive adhesion plaque, suggesting the involvement of integrin *α*v-mediated pathways in TNC-mediated EMT [35]. Additional evidence demonstrated that treatment of breast cancer cells with anti-*α*v integrin neutralizing antibodies, and Src kinase inhibitors abrogates TNC-mediated EMT [35].

 There are four phases involved in the EMT process: the proliferative phenotype of epithelial cells, the epithelial-to-mesenchymal-like cell transition, the motility and migration of the mesenchymal-like cell, and the reversion of EMT through a process called the mesenchymal-to-epithelial transition (MET). Matricellular proteins are likely to be involved in all of these phases of EMT, thus identifying them as novel therapeutic candidates for future drug development.

## 3. Cancer Cell Proliferation

Cancer cells exploit various signaling mechanisms to induce autonomy in tumor growth through the development of self-sufficiency in growth signals, insensitivity to growth-inhibitory (antigrowth) signals, the evasion of programmed cell death (apoptosis), and a limitless replicative potential sustained by angiogenesis, all of which contribute to the uncontrolled proliferation in tumor cells. Stromal cells of the tumor microenvironment play a dynamic role in determining malignancy phenotype. Indeed, the preponderance of evidence has implicated matricellular proteins in the crosstalk between tumor and the surrounding stromal fibroblasts (CAFs) responsible for the acquisition of properties that promote tumor development and metastasis formation.

 In contrast to the role of SPARC in promoting EMT, SPARC expression in ovarian and pancreatic cancer cells decreased tumor growth, increased apoptosis and reduced the ability of cancer cells to induce tumors in nude mice [36–38]. The decreased expression of SPARC in ovarian tumors and pancreatic adenocarcinoma is attributed to the aberrant hypermethylation of the SPARC promoter [37, 39, 40]. SPARC binds to several types of collagen, including collagen I and III, which are the major structural proteins of the ECM produced by host cells in response to the subcutaneously injected tumor cells [40–42]. It was proposed that SPARC exerts its anti-proliferative role in primary and metastatic sites at least in part by increasing the collagen content and mechanical stiffness of the fibers surrounding the tumor, thus restricting the growth of the tumor. However, the role of SPARC in tumor development and metastasis varies because of its context-specific functions (EMT promotion versus inhibition of cell growth) [36]. The disparate effect of SPARC is also evident between stromal fibroblasts and cancer cells. SPARC promotes the proliferation of stromal cells while inhibiting cancer cells [43]. The apparent paradoxical functions of SPARC may arise from the different biochemical properties of the SPARC sources or from the differential responses to SPARC from malignant and stromal cells.

 In glioma cells, CCN1 can enhance tumorigenicity by promoting cell proliferation and survival through integrin *α*v*β*3- and *β*1-activated ILKs to stimulate the *β*-catenin T-cell factor (TCF)/lymphocyte-enhancing factor 1 (LEF-1) and PI3K/PKB signaling pathways [22]. The elevated expression of CCN1 in tumorigenic glioma cell lines accelerates their growth *in vitro* and enhances their anchorage-independent proliferation in soft agar [22]. The suppression of CCN1 by antisense strategy abolishes anchorage-independent growth [22]. The mechanism underlying this phenotype is likely the phosphorylation of glycogen synthase kinase- (GSK-) 3*β*, followed by the subsequent cytoplasmic accumulation and nuclear translocation of *β*-catenin leading to the transcriptional activation of the promitogenic factor cyclin D1 [22]. Moreover, the overexpression of CCN1 also promotes the PKB-mediated inhibition and phosphorylation of the apoptotic effector BAD to impede cell death induced by the caspase cascades [22]. The proproliferative role of CCN1 is further substantiated by its ability to induce large and highly vascularized tumors in nude mice [44]. CCN1 has been shown to regulate breast cancer proliferation and survival by participating in a positive CCN1-*α*v*β*3 autocrine loop; CCN1 stimulates the activation of ERK1/2-MAPK, which increases the expression of integrin *α*v*β*3 expression [44]. CCN1 has been identified as the direct target gene of cAMP-response element-binding protein (CREB) in melanoma, and its expression is negatively regulated by the CREB-mediated inhibition of CCN1 promoter transcription [45].

 TNC promotes cellular proliferation in a variety of cell types, including tumor cells, carcinoma-associated fibroblasts and endothelial cells within the tumor stroma [32]. TNC harbors both adhesive and anti-adhesive sequences that can either support or prevent cell spreading and proliferation [32]. The interplay between TNC, endothelin receptor type A (EDNRA), integrin *α*5*β*1, fibronectin, syndecan-4, and tropomyosin 1 has been reported to particularly contribute to TNC-induced cell proliferation in cancer via the activation of various signaling pathways (see [32]). For instance, TNC was shown to stimulate cancer cell proliferation by inducing EDNRA, the gene encoding the receptor for endothelin-1, and by preventing cells from adhering to fibronectin. This effect probably occurs through the competitive binding of TNC to fibronectin with the integrin *α*5*β*1 coreceptor, syndecan-4, which then blocks the activation of the RhoA protein, FAK, and tropomyocin-1 in the process [46, 47]. Other signaling pathways activated by TNC that contribute to enhanced cancer cell proliferation include the Wnt, TGF-*β* and MAPK signaling pathways [32, 33, 48]. Furthermore, tumor-derived TNC has been demonstrated to promote the survival and outgrowth of pulmonary micrometastases by enhancing the fitness of metastasis-initiating cancer cells through increased expression of stem cell signaling components, such as leucine-rich repeat-containing G protein-coupled receptor 5 (LGR5) and musashi homolog 1 (MSl1) [49]. LGR5 is a target gene of the Wnt pathways, whereas MSl1 is a positive regulator of Notch signaling [49]. These signaling pathways have been implicated in cancer cell proliferation and tumor formation [50, 51]. It is likely that TNC increases the cell-autonomous expression of the Notch and Wnt signaling components to promote the proliferation of cancer cells in primary and metastatic tumors.

OPN has been implicated in tumor growth and metastasis based on studies using gene expression analysis in human and animal tumors, studies using DNA transfection and clinical investigations in human malignancies [52–54]. In particular, higher levels of OPN expression have been reported in three types of lung cancer, including small cell carcinoma, squamous carcinoma, and adenocarcinoma [55]. The downregulation of OPN inhibits the proliferation rate of a human lung adenocarcinoma epithelial cell line and *in vivo *tumor growth by inducing G1-phase cell cycle arrest and instigating late apoptosis and necrosis in these cells [55]. Moreover, the proliferation and invasiveness induced by OPN expression are linked to the different OPN slice variants; OPN-b mainly affects the cell proliferation, whereas OPN-c shows a strong correlation with invasive behavior [55]. Collectively, OPN has been identified as a potential biomarker for proliferation and invasiveness in lung cancer.

 Angiopoietin-like 4 (ANGPTL4) was first described as an adipocytokine that participates in adipogenesis and as an endocrine signal involved in the regulation of lipid and glucose metabolism [56–58]. ANGPTL4 was recently defined as a matricellular protein with a potential role in tumor growth [59, 60]; however, the role of ANGPTL4 in metastasis still remains contradictory. Recently, prostaglandin E_2_ (PGE_2_) and hypoxia were shown to have a synergistic effect on the expression of ANGPTL4 in colorectal cancer [61]. ANGPTL4 enhances colorectal carcinoma cell proliferation through its effects on STAT1 signaling mediated by the MAPK and Src signaling pathways [61]. The clinical relevance of these results is further validated by the findings that the expressions of both ANGPTL4 and STAT1 are elevated in half of the human colorectal cancers tested [61]. ANGPTL4 is also suggested as a diagnostic marker of primary and metastatic sites in clear cell renal-cell carcinoma (ccRCC) [62]. ANGPTL4 expression is widespread among various tumors, and its suppression impairs tumor growth due to the enhanced apoptosis [63]. ANGPTL4 interacts with integrins to stimulate the NADPH oxidase-dependent production of superoxide, thereby triggering the PI3K/PKB*α* and ERK prosurvival pathways as a result of high superoxide to hydrogen peroxide ratio and activated Src signaling [63].

## 4. Tumor Angiogenesis

Angiogenesis is an essential requirement for the metastatic progression and growth of tumors. Apart from nourishing tumors with nutrients and oxygen, the formation of new blood vessels is also needed to aid the tumor cells in exiting the primary tumor site and entering the blood circulation for metastasis at a secondary site [64]. The vascularity of the primary tumor correlates with the formation of metastatic foci that will inherently affect patient prognosis and survival [64]. Tumor-induced angiogenesis is regulated by several angiogenic factors, such as growth factors and adhesion molecules [65–67]. Studies have also shown that ECM, matricellular proteins, and stromal cells are involved in the formation of tumor vasculature [65, 68–70]. The role of some matricellular proteins in tumor angiogenesis remains controversial, as there are various studies supporting both pro- and antiangiogenic effects.

The CCN members were reported to be involved in angiogenesis and tumorigenesis [21, 71, 72]. Earlier reports showed that elevated levels of Cyr61 are associated with advanced breast adenocarcinoma, PDAC, and gliomas, which suggest its involvement in cancer metastasis [22, 23, 25, 73–75]. For instance, Cyr61-knockout mice develop vascular defects that ultimately lead to embryonic death, illustrating the proangiogenic functions of Cyr61 [76]. Cyr61 alone can recapitulate angiogenic events *in vitro* by promoting endothelial cell proliferation, adhesion, migration, survival, and tubule formation through *α*v*β*3 (activation-dependent) and *α*6*β*1-heparin sulfate proteoglycan (activation independent) [77]. Moreover, ectopic expression of Cyr61 in breast cancer-derived tumors induces increased tumor growth and vascularization *in vivo *[25]. Although the downstream intracellular events have yet to be ascertained, the prosurvival effect of Cyr61 on angiogenic endothelial cells in breast cancer may be mediated through the integrin *α*v*β*3/focal adhesion kinase (FAK)/PI3K/PKB signaling pathway [78].

OPN is a secreted matricellular phosphoprotein that mediates angiogenesis by associating with integrin *α*v*β*3 on endothelial cells [79, 80]. Neovascularization was induced *in vivo* by the constitutive overexpression of OPN in murine neuroblastoma and breast cancer cells [81, 82]. OPN has been postulated to upregulate the expression of Cyr61; both OPN and Cyr61 may then interact with the integrin *α*v*β*3 receptors on the surface of endothelial and tumor cells to facilitate angiogenesis (see [83]). The role of TNC in tumor angiogenesis is unequivocal. Its expression has been correlated with the degree of tumor neovascularization in human gliomas and melanoma cells, and tumor cells xenografted into TNC knockout mice demonstrate a regression of tumor growth and angiogenesis [84, 85]. It has been postulated that TNC promotes angiogenesis by acting as a chemoattractant for endothelial cells, initiating endothelial cell differentiation, survival, proliferation involving integrin *α*v*β*3, and vascular endothelial growth factor (VEGF) and also by stimulating VEGFA expression, resulting in endothelial cell migration, proliferation and the subsequent formation of capillaries in the tumor [84, 86].

 The thrombospondin (TSP)-1 and -2 family of matricellular proteins are inhibitors of angiogenesis [87–89]. In fact, their antiangiogenic function has incited interest as them being anti-tumor agents [87]. TSP1 and TSP2 have been demonstrated to suppress angiogenesis by inhibiting endothelial cell migration, inducing endothelial cell apoptosis and preventing the interaction of growth factors with the cell surface receptors of the endothelial cell [87–91]. Tumor cells with activated Ras can increase the level of myc phosphorylation through the activation of the PI3K/Rho pathway, which results in the inhibition of TSP1 gene expression, creating an immediate pro-angiogenic tumor microenvironment [92]. This pro-angiogenic effect in the tumor may also be partly regulated by the loss of the p53 tumor suppressor genes [93, 94]. Unlike the low expression level of TSP1 in the tumor cell, adjacent stroma fibroblast cells can secrete high levels of TSP1, suppressing angiogenesis and tumor growth [95]. TSP1 can induce endothelial apoptosis and inhibit angiogenesis via the sequential activation of CD36, Src family tyrosine-protein kinase (Fyn), caspase-3, and the p38 MAPK cascade to curtail tumor growth [96, 97]. Conversely, a limited number of studies also showed an angiogenic phenotype for these proteins. One study showed that TSP1 induces a concentration-dependent outgrowth of microvessels from rat aortic rings [87, 98]. Recently, TSP1 was shown to induce neovascularization in quail chorioallantoic membranes and in matrigel plug formation assays via its interaction with integrin *α*9*β*1 on endothelial cells [87, 99]. Although none of the studies point to a role of TSP1 and TSP2 in tumor angiogenesis, such a postulation cannot and should not be ruled out.

 Similarly, SPARC possesses pro- and antiangiogenesis phenotypes [36]. Although shown in limited studies as pro-angiogenic, it is more often known as an antiangiogenic agent and, thus, has sparked great enthusiasm in its therapeutic potential for vascularized tumors [100–104]. The overexpression of SPARC blocks angiogenesis both *in vitro* and *in vivo* in neuroblastoma [105]. SPARC overexpression also led to a significant decline in microvessel density, delayed tumor formation, and reduction of tumor size in hepatocellular carcinoma xenografts [106]. SPARC inhibits angiogenesis via a variety of molecular pathways, such as interacting directly with angiogenic VEGF to prevent interaction with its cognate receptor, thus preventing endothelial cell proliferation. SPARC also indirectly inhibited angiogenesis by regulating angiogenesis-related genes, such as matrix metalloproteases (MMPs) [107]. On a translational level, two antiangiogenic SPARC peptides have recently been shown to inhibit the progression of neuroblastoma tumors both *in vitro* and *in vivo*, heralding an optimistic future for SPARC as an antivascular cancer therapeutic [108].

Recently identified as a tumor-secreted pro-metastasis factor and as a matricellular protein, ANGPTL4 is upregulated in many epithelial tumors [63, 109–111]. More importantly, it has been proposed to be a pro-angiogenic factor that promotes neovascularization [112, 113]. ANGPTL4 was shown to protect endothelial cells from apoptosis, promote *in vitro* the tube formation of endothelial cells and promote angiogenesis in an *in vivo* mouse model, suggesting a diverse role for ANGPTL4 in metastasis [112, 113]. Although largely acknowledged as a pro-angiogenic factor, a small number of studies also portrayed ANGPTL4 as an antiangiogenic factor, thereby preventing metastasis [114, 115]. The reason for this discrepancy is still unclear; however, recent discussions have suggested that the difference may be attributed to the position of the fusion tag in the recombinant ANGPTL4 used in the various studies [116].

It is clear that angiogenesis is a “life-line” for tumors. Without the formation of new vascular niches to provide continuous blood flow and nutrients, most primary tumors will not be able to grow beyond 1-2 mm^3^ and/or metastasize to secondary sites [117]. Given the myriad molecular roles that matricellular proteins participate during tumor angiogenesis (or antiangiogenesis), these proteins definitely warrant additional research to elucidate whether they are pharmacological targets or agents. The addition of matricellular proteins into the repertoire of antivascular strategies will hopefully address some challenges in this aspect of tumor biology and provide a viable alternative treatment option for cancer and other vascular diseases.

## 5. Cancer Metastasis

The acquisition of migratory abilities in cancer cells is a prerequisite for successful execution of the metastasis process. Metastasis is a multiple process, beginning with local tumor invasion, followed by intravasation by cancer cells into blood and lymphatic vessels, the transit of cancer cells through the circulatory system, and the exiting of cancer cells by extravasation into distant tissue for colonization [118]. This migratory ability is often integrin-mediated and is positively correlated with tumor metastasis [119–121]. Integrins expressed on the surface of cancer cells are important in all these processes, as they are needed for cell adhesion via interaction with many ECM molecules including fibronection, other matrix proteins, and matricellular proteins [119].

The role of TSP in cancer metastasis is complex; various studies suggested a dual role of TSP1 acting as an adhesive protein and a modulator of extracellular proteases to promote tumor invasion (see review [122]). The expression level of TSP1 in metastatic cells is lower than in non-metastatic or normal cells [123–125]. Notably, cancer patients with low TSP1 expression have increased recurrence and lower survival rates [126]. Breast carcinoma cells lacking the adhesive motif on TSP1 displayed a suppressive effect on tumorigenesis [127]. The cell surface receptor CD47 binds to the adhesive motif Val-Val-Met on the C-terminal domain of TSP1 [128]. The elevated expression of CD47 can stimulate *β*1 integrin-mediated cancer cell motility via the inhibition of ERK activity and the suppression of cyclic AMP levels [96, 129]. During metastasis, cancer cells reduce their adhesion, detach from the primary tumor site and invade the surrounding tissue through the cleavage of ECM by proteolytic activity. Early studies demonstrated that TSP1 upregulates plasmin, a proteolytic enzyme that degrades ECM, thus aiding cell invasion [130]. At high expression levels, TSP1 activates the plasminogen/plasmin system and therefore promotes cell invasion; however, reduced TSP1 expression levels promote the interaction between cancer cells and matrix necessary for tumor growth [130, 131].

Similarly, TNC also contains both adhesive and anti-adhesive sequences that could either support or prevent cell spreading [32]. TNC accumulates at the invasive front of TNC-secreting cells, such as carcinoma cells and cancer-associated fibroblasts (CAFs) [32, 95]. A mechanistic study showed that TNC promotes colon carcinoma cell invasion via the epidermal growth factor receptor (EGFR) and hepatocyte growth factor (HGF) signaling pathways; TNC secreted by CAFs activates EGFR and HGF [132]. HGF binds to its cognate receptor c-Met and triggers Rac activation, while EGFR signaling inhibits RhoA activation, consistent with the phenotypical signaling pathways in migratory cells [132]. TNC also induced cell migration via its interaction with promigratory factors (lysophosphatidic acid/platelet derieved growth factor) together with PI3K, RhoKinase (ROCK), and MAPK/ERK kinase (MEK) pathways [133, 134].

The expression of SPARC is induced in tissues undergoing repair in response to cellular injury and in epithelia with a high ECM turnover [135, 136]. SPARC expression is associated with aggressive invasion and metastasis in prostate cancer, glioma, and melanoma cells [136–140]. The inhibition of SPARC expression diminishes tumorigenicity and metastatic dissemination of these cancer cells [102, 141]. SPARC interacts with different ECM components, including collagens, laminin, fibronectin, and vitronectin [135, 142, 143]. It functions as a counter-adhesive agent by inducing cell rounding and disassembly of focal adhesion contacts in normal endothelial cells, most likely by antagonizing the integrin-ECM interaction [5]. SPARC also influences the secretion and activation of several MMPs, including MMP-1, -2, and -7, to promote cancer invasiveness [107, 144, 145]. The increase in migration generated in response to SPARC was mediated through the activation of integrins *α*v*β*3 and *α*v*β*5 [13]. However, the SPARC protein does not contain a RGD sequence; thus, the SPARC-induced motility of cancer cells likely involves an indirect mechanism [12]. There is ample evidence suggesting that SPARC regulates integrin signaling and the ability of integrins to interact with structural components of the ECM by influencing the activation of ILK [12, 146, 147]. SPARC reduces the surface localization and clustering of integrin subunits *α*v, *β*1, *β*3, and *β*5 [148]. Altogether, these findings reveal that SPARC influences integrin clustering and activation as well as its ability to interact with ECM components. It is likely that the diverse effects of SPARC on tumor invasiveness are elicited by its ability to control the pleiotropic interactions and functions of integrins.

The expression of Cyr61 is elevated in advanced breast adenocarcinoma, pancreatic cancer, gastric cancer, osteosarcoma, and gliomas, among others [22–25]. Cyr61 promotes cancer cell motility and invasiveness through cyclooxygenase-2 (COX-2) upregulation via *α*v*β*3/NF-*κ*B-dependent pathways [149]. In addition, Cyr61 also promotes the upregulation of chemokine receptors CXCR1 and CXCR2 through the integrin *α*v*β*3/Src/PI3K/Akt pathway, which is involved in the transendothelial migration of gastric cancer cells [150]. Cyr61 released by cancer cells in hypoxic conditions is proposed to induce the activation of plasminogen activator inhibitor-1 (PAI-1); however, it also improved the invasion ability of cancer cells [151]. It appears to be contradictory that PAI-1 is required for invasion because elevated PAI-1 should reduce plasmin generation from plasminogen. It was believed that tumor invasion requires the cooperation between both proteolytic and inhibitory activities, most likely to control and confine the areas of invasive growth. It has been suggested that the role of PAI-1 may be merely to protect tumors from ongoing urokinase-type plasminogen activator-mediated proteolysis while invading the ECM [152].

ANGPTL4 is one of the most highly predictive genes associated with breast cancer metastasis to the lung, and it is highly upregulated in ccRCC and oral tongue squamous cell carcinoma [109, 113, 153]. The elevated ANGPTL4 expression in many highly metastatic epithelial tumors suggests that ANGPTL4 is a critical mediator of the transmigration process [63, 109–111]. Recent studies demonstrated that ANGPTL4 expression in hepatocellular carcinoma can promote transendothelial migration via the upregulation of vascular cell adhesion molecules-1 (VCAM-1)/integrins *β*1 signaling [154]. Consistently, TGF-*β*, which upregulates ANGPTL4 in breast tumor cells, caused enhanced vascular leakiness and promoted the transendothelial migration of breast tumor cells to the lung [155]. In addition, tumor-secreted ANGPTL4 can disrupt the junction between adjacent endothelial cells by interacting with integrin *α*5*β*1 to activate the Rac/PAK signaling, followed by interaction with VE-cadherin and claudin-5 [116]. This results in the internalization and declustering of the tight and adherens junction proteins on the endothelial cells, leading to the disruption of the vascular barrier that is believed to allow invasion by the cancer cells [116]. Furthermore, clinical studies have correlated ANGPTL4 expression to venous and lymphatic invasion in human gastric and colorectal carcinoma, which further emphasizes the role of ANGPTL4 in tumor metastasis [110]. In addition, elevated ANGPTL4 was shown to increase cell migration via the interaction with vitronectin and fibronectin in the ECM as well as integrins *β*1 and *β*5 on the cell surface [59, 60]. The former interaction regulates the availability of the local ECM, whereas the latter activates the integrin-mediated intracellular signaling necessary for tumor cell motility [59, 60]. Together these studies implicate tumor-derived ANGPTL4 in cancer metastasis via its effect on endothelial integrity and cellular migration. Given the multitude of evidence pointing to a role for ANGPTL4 in tumorigenesis, elucidating other molecular mechanisms necessary for cancer progression that are induced by this protein is worthwhile.

Given the myriad ways in which these matricellular proteins contribute to cancer metastasis, it is likely that they act cooperatively with other matricellular proteins to induce cancer cell invasion. Studies on the interactive role among these matricellular proteins are limited and further investigation is necessary to design molecularly targeted anti-metastatic therapy.

## 6. Evade Immune Surveillance and Anoikis Resistance

Apoptosis is a determinant factor modulating metastasis efficiency. As a barrier to metastases, cells normally undergo apoptosis after they lose contact with the extracellular matrix (ECM), a cell-death process termed anoikis [156, 157]. The ability to survive in the absence of normal matrix components (anoikis resistance) represents a crucial property of metastatic cells that permits malignant tumor cells to survive at crucial steps in the metastasis pathway [157]. The acquisition of anoikis resistance is a prerequisite for the initial step of metastatic dissemination that requires the detachment of epithelial tumor cells from the ECM [157]. Anoikis resistance is also required for cancer cell survival while traveling through the lymphatic and circulatory systems into the secondary tissue sites [157]. Circulating tumor cells also devise immunosuppressive strategies to avoid patrolling innate immune cells, such as natural killer cells, macrophages, or adaptive immune responses involving B and T lymphocytes [158]. These immunosuppressive strategies would increase tumor tolerance against immune responses and improve their survival capabilities. In recent years, members of matricellular proteins were shown to be involved in different aspects of the inflammatory response and cancer cell survival during tumor development; therefore, a better understanding of the molecular mechanisms underlying anoikis resistance and immunosuppressive strategies would offer novel anticancer treatments.

 Metastatic tumors are characterized by the overproduction of several matricellular proteins that are believed to play a key role in more advanced steps of tumor progression, such as tumor induced angiogenesis, the inhibition of infiltrating immune cells and the protection of disseminated tumor cells in circulation [156, 158, 159]. An increase in SPARC activity is associated with diminished immune surveillance during tumorigenesis [160]. In particular, melanoma cells with suppressed SPARC expression resulted in enhanced polymorphonuclear leukocyte (PMN) recruitment, a first-line of defense in the immune surveillance against cancer, and in increased antitumor cytotoxic activity against tumor growth *in vitro* and *in vivo *[102, 160]. Furthermore, the *in vivo* depletion of PMN was able to reverse tumorigenesis of SPARC-deficient melanoma cells, suggesting that SPARC can inhibit the recruitment of PMN to the tumor environment [161]. Recent works highlighted that SPARC may mediate its action by integrin-dependent signaling. SPARC deficiency can induce the production of fibronectin [12]. Fibronectin activates integrin via the ILK activity and triggers the production of specific chemoattractants for PMN, such as GRO, IL8, and leukotriene [12, 146, 162–164].

OPN is highly expressed in chronic inflammatory diseases and possesses chemotactic activity for macrophages and neutrophils [165–167]. Elevated OPN expression in breast, myeloma, and prostate cancers is associated with poor prognosis [168–170]. OPN is secreted by host stromal cells and cancer cells; however, the role of OPN in metastasis is dependent on the site of production. Host-derived OPN (hdOPN) in the ECM is in an aggregated form. hdOPN could be antimetastatic by acting as a macrophage chemoattractant, resulting in the inhibition of tumor growth, and survival [165]. OPN produced by endothelial cells was shown to promote angiogenesis and therefore favor metastasis [83]. In contrast, tumor-derived OPN (tdOPN) is in a soluble form and acts as an inhibitor of macrophage functions, thus promoting the metastatic process by supporting the growth, invasiveness and survival of tumor cells in the circulation [171, 172]. Early work showed that tdOPN inhibited macrophage cytotoxicity against tumors, promoting tumor dissemination [173]. Interestingly, OPN secreted by macrophages inhibited tumor growth [174, 175]. Thus, these observations warrant further investigation to determine the precise role of specific cell-type, derived OPN and the contribution towards tumor progression.

TNC expression is increased in cancer and noncancerous inflammatory diseases [32, 49]. Early studies on tumor biopsies showed an inverse correlation between a higher density of macrophagic/microglial infiltrates and TNC expression in a malignancy group of glioblastomas, suggesting that TNC may play a crucial role in regulating the infiltration of the monocyte lineage in human glioma [176]. Indeed, other studies also showed increased expression of TNC correlates with the recurrence of NSCLC, where it inhibits the effector functions of tumor-infiltrating lymphocytes [177]. The stromal compartment of TNC null mice contained significantly more monocytes/macrophages than the tumor stroma of wild-type mice, suggesting that TNC might promote tumor growth while at the same time blocking the inflammatory infiltrates [178]. Furthermore, TNC inhibited *in vitro* T-lymphocyte activation by blocking integrin *α*5*β*1- and *α*4*β*1-mediated cell adhesion to fibronectin, similar to the effect of SPARC on PMN via integrin-dependent signaling [179, 180].

Early studies demonstrated that TSP1 produced by squamous epithelial cells and monocytes plays a role in monocyte-mediated killing of cancer cells [181]. The overexpression of TSP1 in melanoma cells enhanced macrophage recruitment into xenograft tumors grown in immunodeficient mice and polarized macrophages to the M1 antitumorigenic phenotype [182]. Consistent with the recruitment of macrophages, TSP1 null mice exhibited a reduction in inflammatory responses with a decrease in macrophage recruitment [183]. Furthermore, TSP1 can inhibit T cell receptor signal transduction and induce anergy in activated T cells via CD47 [184, 185]. More recently, TSP1 was shown to possess the capacity to generate regulatory T cells (TREG) from naive T cells through CD47 [186]. Metastatic melanoma secreted TSP1 or blocking of CD47 attenuated the induction of TREGs by melanoma while induced TREGs were able to actively suppress the immune response [187]. Furthermore, TSP1 can activate latent TGF-*β*, and the activation of this immunosuppressive cytokine induces more TREGs in the tumor microenvironment [188]. Interestingly, SPARC can inhibit the production of TSP1 in endothelial cells; however, the effects of SPARC on the expression of TSP1 in tumor remain elusive.

Anoikis resistance is essential for the survival of metastatic tumor cells. A recent study demonstrated that ANGPTL4 interacts with integrins to stimulate a redox-based prosurvival pathway that confers resistance against anoikis [63]. ANGPTL4-activated integrins increase O_2_^−^ generation and stimulate downstream PI3K/AKT and ERK1/2 survival pathways to confer anoikis resistance [63]. Specific neutralizing antibodies against ANGPTL4 that antagonize the interaction between ANGPTL4 and integrins result in a dose-dependent reduction in the O_2_^−^ : H_2_O_2_ ratio and increased apoptotic cancer cells. ANGPTL4 deficiency in cancer cells also abolished the tumorigenic abilities of these cells in athymic nude mice [63].

It is evident that metastatic tumor invasion to distal sites can be impaired by modulating the ability of tumor cells to evade immune surveillance or its survival from anoikis. Therefore, it is tempting to speculate that targeting these matricellular proteins (e.g., SPARC, ANGPTL4, and hdOPN) would either increase the infiltration of immune cells to inhibit tumor growth or induce apoptosis by stripping off their anoikis survival capabilities. Future work is eagerly awaited to validate this speculation because this finding might open interesting therapeutic perspectives even to cancer patient diagnosed with metastatic disease.

## 7. Therapeutic Exploitation and Conclusion

Despite the multitude of studies implicating matricellular proteins in the metastatic cascade, the challenge still exists for researchers to decipher more matricellular protein-interacting partners and the molecular signaling mechanisms in the midst of numerous confounding factors that occur during tumor metastasis (Table 1). Although the activation of the PI3K/PKB, ERK1/2 MAPK, and NF-*κ*B signaling pathways by diverse matricellular proteins appears to be a recurrent theme by which matricellular proteins modulate tumor survival and growth, there is still much to elucidate on how exactly these matricellular proteins modulate and orchestrate the entire metastastic process. Nevertheless, given their multifaceted roles in tumorigenesis, matricellular proteins should be considered strong potential therapeutic targets in cancer treatment (Figures 1 and 2). Indeed, several clinical studies have already established the use of matricellular proteins as prognostic markers for tumor progression [72, 189, 190]. To date, various therapeutic approaches to either increase or reduce the levels of matricellular proteins are being developed for cancer treatment. These approaches include cell-based gene therapy and the systemic delivery of neutralizing antibodies, recombinant proteins, or synthetic peptides.

 In cell-based gene therapy, a recombinant adeno-associated virus mediates the delivery of a vector expressing either the matricellular protein of interest or selected modules of the matricellular protein involved in the cell adhesion molecule interaction. For instance, the delivery of adenovirus encoding the type III repeat domain of TSP1 inhibited proliferation and induced apoptosis of a melanoma cell line *in vitro *and *in vivo *[191]. Blocking the integrin receptors by RGD peptidomimetic agents or the peptide fragment of CD44 may be used to interfere with their respective OPN interactions, impairing tumor motility and survival [192, 193]. Additionally, the ablation of SPARC transcription using antisense RNA has successfully inhibited human melanoma and gastric cancer growth in nude mice [102, 194]. Recently, two antiangiogenic SPARC peptides have recently been shown to inhibit the progression of neuroblastoma tumors both *in vitro* and *in vivo*, heralding an optimistic future for SPARC as an antivascular cancer therapeutic [79]. The use of neutralizing antibodies against target matricellular proteins expressed in tumors could also be a viable therapeutic option. Recently, the use of a monoclonal antibody against ANGPTL4 caused a significant retardation in the growth of melanoma in a murine model via a redox-based apoptotic pathway [63].

 Matricellular proteins reside at the crossroads of cell-matrix communication and can serve as modulators for regulatory networks of metastasis and tumorigenesis. Matricellular proteins act to provide signals that influence cell activities characteristic of the metastatic cascade, such as cell proliferation, migration, survival, angiogenesis, EMT, and the maintenance of specialized stem cell niches. Owing to these properties, the manipulation of matricellular proteins for the adjuctive or multimodal therapeutic anti-tumor treatments may fare better in treating cancer than therapies that target one event. Therefore, a more detailed understanding of matricellular proteins and their respective cell adhesion signaling pathways is critical not only to advance basic oncology knowledge but also for the design of new anticancer therapeutic strategies.

## Figures and Tables

**Table 1:** Overview of the marticellular protein cell-adhesion signaling pathways and their biological and clinical implications.

Matricellular protein	Cell adhesion partner(s)	Signaling pathways	Cellular and biological effects	Clinical implications
ANGPTL4	Vitronectin, Fibronectin, Integrin *β*1, *β*5	TGF*β* via smad signaling, redox-based pro-survival via PI3K/PKB and ERK1/2 downstream survival pathways	Regulates ECM availability, cell migration, angiogenesis confers anoikis effect on tumor cells	Wound repair, cancer metastasis

Cyr61	Integrin *α*2*β*1, *α*6*β*1, *α*D*β*2, *α*M*β*2, *α*IIb*β*3, *α*v*β*3, *α*v*β*5 Syndecan-4, perclean	PI3K/PKB, ERK1/2, MAPK, NF-*κ*B signaling pathways	Promotes cell proliferation, motility, survival, invasiveness confers anti-apoptotic phentotype	Cancer metastasis and tumorigenesis

OPN	CD44, integrin *α*v*β*1, *α*v*β*3, and *α*v*β*5	NF-*κ*B,VEGF signaling, Src-mediated “inside-out” signaling, integrin-linked ILK, and PKB survival pathway	Integrin-mediated cancer cell migration, angiogenesis, inhibition of apoptosis ECM degradation via MMPs	Cancer metastasis

SPARC	Integrin *α*5*β*1	PKB prosurvival pathway		Cancer metastasis

TNC	Fibronectin, syndecan-4	MAPK, Wnt, TGF*β*, EGFR, HGF, c-Met signaling pathways	Induction of TNC expression, cell proliferation, migration, invasion Downregulation of DKK-1, increased express and nuclear accumulation of *β*-catenin	Cancer metastasis

TSP1	Integrin *α*3*β*1, *α*4*β*1, *α*6*β*1, *α*v*β*3 CD 47, CD36	Fyn, capase-3, and p38 MAPK, inhibit eNOS/NO signaling, inhibit VEGF/VEGFR2 signaling pathways	Inhibit endothelial cell migration to reduce angiogenesis, modulate level of sGC and cGMP-dependent protein kinase in endothelial cells	Inhibit metastasis via its antiangiogenic phenotype
